# *Nocardia macrotermitis* sp. nov. and *Nocardia aurantia* sp. nov., isolated from the gut of the fungus-growing termite *Macrotermes natalensis*

**DOI:** 10.1099/ijsem.0.004398

**Published:** 2020-08-20

**Authors:** René Benndorf, Jan W. Schwitalla, Karin Martin, Z. Wilhelm de Beer, John Vollmers, Anne-Kristin Kaster, Michael Poulsen, Christine Beemelmanns

**Affiliations:** ^1^​ Leibniz Institute for Natural Product Research and Infection Biology e. V., Hans-Knöll-Institute, Beutenbergstraße 11a, 07745 Jena, Germany; ^2^​ Department of Microbiology and Plant Pathology, Forestry and Agriculture Biotechnology Institute, University of Pretoria, 0028 Hatfield, South Africa; ^3^​ Institute for Biological Interfaces (IBG 5), Karlsruhe Institute of Technology, Hermann-von- Helmholtz-Platz 1, 76344 Eggenstein-Leopoldshafen, Germany; ^4^​ University of Copenhagen, Department of Biology, Section for Ecology and Evolution, Universitetsparken 15, 2100 Copenhagen East, Denmark

**Keywords:** *Macrotermes natalensis*, *Nocardia*, termite gut

## Abstract

The taxonomic positions of two novel aerobic, Gram-stain-positive Actinobacteria, designated RB20^T^ and RB56^T^, were determined using a polyphasic approach. Both were isolated from the fungus-farming termite *Macrotermes natalensis*. Results of 16S rRNA gene sequence analysis revealed that both strains are members of the genus *Nocardia* with the closest phylogenetic neighbours *Nocardia miyunensis* JCM12860^T^ (98.9 %) and *Nocardia nova* DSM44481^T^ (98.5 %) for RB20^T^ and *Nocardia takedensis* DSM 44801^T^ (98.3 %), *Nocardia pseudobrasiliensis* DSM 44290^T^ (98.3 %) and *Nocardia rayongensis* JCM 19832^T^ (98.2 %) for RB56^T^. Digital DNA–DNA hybridization (DDH) between RB20^T^ and *N. miyunensis* JCM12860^T^ and *N. nova* DSM 44481^T^ resulted in similarity values of 33.9 and 22.0 %, respectively. DDH between RB56^T^ and *N. takedensis* DSM44801^T^ and *N. pseudobrasiliensis* DSM44290^T^ showed similarity values of 20.7 and 22.3 %, respectively. In addition, wet-lab DDH between RB56^T^ and *N. rayongensis* JCM19832^T^ resulted in 10.2 % (14.5 %) similarity. Both strains showed morphological and chemotaxonomic features typical for the genus *Nocardia *, such as the presence of *meso*-diaminopimelic acid (A_2_pm) within the cell wall, arabinose and galactose as major sugar components within whole cell-wall hydrolysates, the presence of mycolic acids and major phospholipids (diphosphatidylglycerol, phosphatidylethanolamine, phosphatidylinositol), and the predominant menaquinone MK-8 (H_4_, ω-cyclo). The main fatty acids for both strains were hexadecanoic acid (C_16 : 0_), 10-methyloctadecanoic acid (10-methyl C_18 : 0_) and *cis*-9-octadecenoic acid (C_18 : 1_ ω9*c*). We propose two novel species within the genus *Nocardia *: *Nocardia macrotermitis* sp. nov. with the type strain RB20^T^ (=VKM Ac-2841^T^=NRRL B65541^T^) and *Nocardia aurantia* sp. nov. with the type strain RB56^T^ (=VKM Ac-2842^T^=NRRL B65542^T^).

## Introduction

Members of the genus *Nocardia* are characterized as Gram-positive, non-motile, aerobic bacteria that form a branched mycelium which is easily fragmented forming rod to coccoid-like structures [1]. The genus was established by Trevisan in 1889 [2]. They form a distinct clade within the class *Actinobacteria* together with *Corynebacteriaceae* and *Mycobacteriaceae* due to the presence of mycolic acids in the cell membrane [3].

Like members of these two families, strains of *Nocardia* have been mostly recognized as pathogens of humans, plants and animals [4–7]. Nonetheless, they were also isolated from soil [8] and as symbionts of plants and marine sponges [9, 10]. In light of these studies, biochemistry- and pharmacology-driven studies have shown that *Nocardia* species harbour an enormous biosynthetic potential to produce structurally unique natural products with antiviral, antifungal, antibacterial and immunosuppressive functions [11–14].

We have recently focused on the phylogenetic and chemical characterization of Actinobacteria associated with fungus-growing termites [15], which are terrestrial eusocial invertebrates that occupy most available habitats in (sub)tropical regions where they contribute up to 20 % of carbon mineralization in savannah ecosystems [16–19]. Microbial profiling studies of fungus-growing termite species showed that the core community of the termite gut was distinct from those of the lower and higher non-fungus-growing termites, which suggested an adaptation to different nutritional environments in the host gut [20]. Building on microbial profiling studies, we pursued in parallel a cultivation-based approach to analyse the microbial diversity of fungus-growing termite systems [15]. Here, we describe the isolation of two new *Nocardia* species isolated from the gut of fungus-growing termite *Macrotermes natalensis*.

## Isolation and ecology

In February 2015, termite workers of the genus *Macrotermes natalensis* were collected from a termite colony Mn160 (25° 44′ 34.7″ S 28° 15′ 38.7″ E, Pretoria, South Africa) and actinobacterial strains RB20^T^ and RB56^T^ were isolated from termite guts as previously described [15]. Chitin agar plates supplemented with 0.05 g l^−1^ cycloheximide were incubated aerobically for 21 days at 30 °C and checked daily for the appearance of colonies. Single colonies were transferred onto International *Streptomyces* Project (ISP) 2 medium. The isolated pure cultures of RB20^T^ and RB56^T^ were maintained on ISP2 at 30 °C and as glycerol suspensions (25%, v/v) at −80 °C.

## 16S rRNA gene phylogeny

Genomic DNA extraction, genome sequencing, PCR amplification and sequencing of the 16S rRNA genes of RB20^T^ and RB56^T^ were carried out as previously described [20]. Additionally, sequences of the 16S rRNA genes of RB20^T^ and RB56^T^ were extracted from whole genome data (accession no. WEGK00000000, WEGI00000000.1) using Artemis [21]. blastn analysis was determined using the NCBI database and the results indicated that strains RB20^T^ and RB56^T^ were members of the genus *Nocardia *. The 16S rRNA gene sequences of selected *Nocardia* reference strains were downloaded from the LPSN database (date of access: 2 March 2020) [22] and pairwise sequence similarities were calculated as recommended by Meier-Kolthoff *et al*. [23] on the GGDC web server [24, 25]. The sina sequence alignment service was used to generate 16S rRNA gene sequence alignments [26]. Phylogenetic trees were reconstructed with mega version 7.0.26 [27] using the neighbour-joining (NJ) [28] and maximum likelihood (ML) [29] algorithms. The evolutionary distance model of Tamura [30] was used to generate evolutionary distance matrices for the algorithms with deletion of complete gaps and missing data. For the ML algorithm, discrete Gamma distribution was used (*+*G) and the rate variation model allowed for some sites to be evolutionarily invariable (*+*I). For the NJ algorithm, rate variation among sites was modelled with a gamma distribution. The reliability of the tree topology was evaluated by bootstrap analysis with 1000 resamplings [31].

Strain RB20^T^ shared highest 16S rRNA gene similarity with *Nocardia miyunensis* 117^T^ (=JCM12860^T^; 98.9 %) [32], *Nocardia nova* DSM44481^T^ (=JCM6044^T^; 98.5 %) [33], *Nocardia niigatensis* IFM330^T^ (=NBRC100131^T^; 98.4 %) and *Nocardia pseudobrasiliensis* DSM44290^T^ (=NBRC108224^T^; 98.3 %) [34]. Strain RB56^T^ shared highest 16S rRNA similarity with *Nocardia takedensis* DSM44801^T^ (=MS1-3^T^=NBRC 100417^T^; 98.3 %) [35], *Nocardia pseudobrasiliensis* DSM44290^T^ (=NBRC108224^T^; 98.3 %) and *Nocardia rayongensis* JCM19832^T^ (=RY45-3^T^; 98.2 %) [36]. Lower levels of 16S rRNA gene sequence similarity (<98.2 %) were found to all other type strains of *Nocardia* species (Table S1 and S2, available in the online version of this article).

**Fig. 1.**
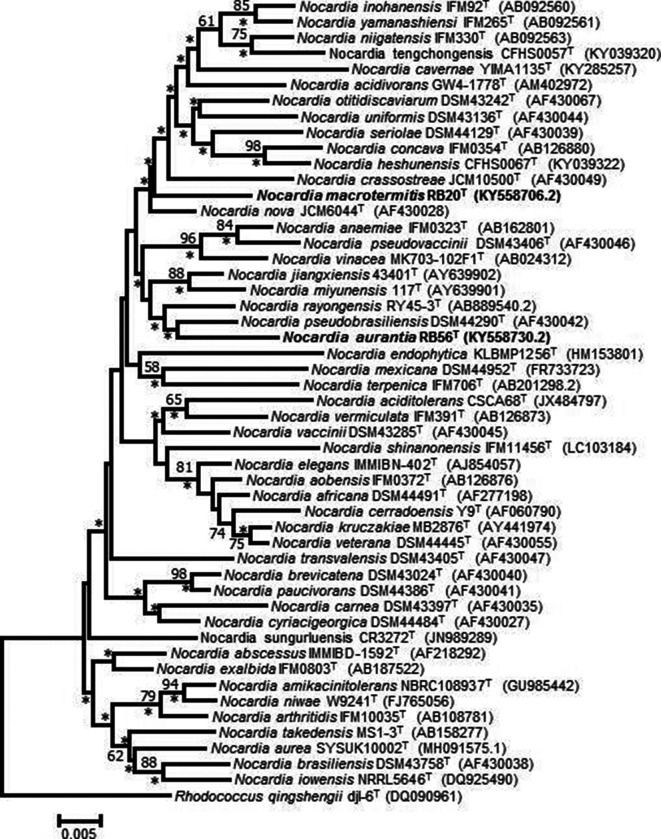
Neighbour-joining tree based on almost-complete 16S rRNA gene sequences showing the relationship between strain RB20^T^ and RB56^T^ and species of the genus *Nocardia. Rhodococcus qingshengii* djl-6^T^ was used to root the tree. Asterisks donate branches that were also recovered in the maximum-likelihood tree (Fig. S1). Only bootstrap values above 50 % (1000 pseudoreplications) are shown. Bar, 0.005 substitutions per nucleotide position.

Phylogenetic analysis using ML and NJ trees indicated that strain RB20^T^ formed a cluster with a larger clade containing *N. nova* JCM6044^T^. Strain RB56^T^ clustered with *N. rayongensis* RY45-3^T^ and *N. pseudobrasiliensis* DSM44290^T^ (Figs 1 and S1). However, the bootstrap support for the topology of this cluster was very low. Based on the analyses of the 16S rRNA gene sequence similarities and phylogenetic trees, *N. miyunensis* 117^T^, *N. nova* DSM44481^T^, *N. pseudobrasiliensis* DSM44290^T^ and *N. rayongensis* JCM19832^T^ were selected as reference strains.

## Genome features

The DNA G+C content of the genomic DNA was determined from the whole genome sequences [23, 37]. DNA–DNA hybridization (DDH) was performed by the Deutsche Sammlung von Mikroorganismen und Zellkulturen (DSMZ) identification service as a classical wet-lab experiment. The required DNA was obtained as described by Cashion *et al.* [38] and the DDH experiments were performed in duplicate according to the methods of De Ley *et al.* [39] and Huss *et al.* [40]. Furthermore, DDH analysis was performed *in silico* using genomes deposited at public databases (Tables S3 and S4) [41].

It was recommended by Stackebrandt and Ebers [42] that a 16S rRNA gene sequence similarity range above 98.7–99.0 % requires additional genome analysis to prove the genomic uniqueness of novel isolates. To confirm the novel species status, we then compared DNA–DNA similarities of the closest type strains of the closest species of the genus *Nocardia* (* N. miyunensis* JCM12860^T^ [32], *N. nova* DSM44481^T^ [33], *N. takedensis* DSM44801^T^ [35], *N. pseudobrasiliensis* DSM44290^T^ [34], *N. rayongensis* JCM19832^T^ [36] and our isolates. First, digital DDH (dDDH) values were determined for RB20^T^ and the closest relatives, *N. miyunensis* JCM12860^T^ and *N. nova* DSM44481^T^, resulting in 33.9 and 22.0 %, respectively (Table S3). For strain RB56^T^ and the closest relatives *N. takedensis* DSM44801^T^ and *N. pseudobrasiliensis* DSM44290^T^ the dDDH values were 20.7 and 22.3 %, respectively (Table S4). Wet-lab DDH was performed for RB56^T^ and *N. rayongensis* JCM19832^T^ due to the lack of whole genome sequence data for *N. rayongensis* JCM19832^T^ and resulted in a DDH value of 14.5 % (10.2 %). In both cases, the obtained values are below the threshold value of 70 % for the definition of bacterial species recommended by Wayne *et al.* [43].

Genome analysis of RB20^T^ and RB56^T^ showed that both strains had a similar genome size of approximately 8.6 Mb with 60 contigs for RB20^T^ and 67 contigs for RB56^T^ (Table S5) N50 size of RB20^T^ is 425 626 bp and for RB56^T^ 451 059 bp. Total CDS were 7454 and 7605 for RB20^T^ and RB56^T^ and the genomes had a completeness of 98.9 and 99.7 %. The G+C content was 67.2 % for RB20^T^ and 69.4 % for RB56^T^, which is typical for this genus (64–72 %) [1].

## Physiology and chemotaxonomy

For chemotaxonomic analyses, freeze-dried cells were obtained from culture grown in ISP2 for 3 days at 28 °C on a rotary shaker at 180 r.p.m. The diagnostic diamino acid of the cell wall was determined in whole-cell hydrolysates by paper chromatography according to Hasegawa [44]. Whole-cell sugars were examined according to Schumann [45]. The occurrence of free mycolic acids was determined by TLC as described by Minnikin [46]. Respiratory quinones of the strains were extracted and separated as described by Collins *et al.* [47] and identified as described by Wink *et al.* [48]. To verify the occurrence of the menaquinone MK-8 (H_4, ω-cyclo_) type strains of *N. asterioides* (IMET 7547^T^) [49, 50] and *N. carnea* (IMET 7504^T^) producing this menaquinone were analyzed in parallel. Polar lipids were extracted by the method described by Minnikin [51] and identified by two-dimensional thin-layer chromatography as described by Collins and Jones [52]. Extraction and analysis of fatty acids was done by the DSMZ Identification service by described standard methods [53]. The glycolysation of the muramic acid of the peptidoglycan was analyzed as described by Schumann [45]. The reference strain investigated in parallel was *Rhodococcus rhodochrous* IMET 7374^T^ containing glycolyl and *Nocardoides albus* IMET 7807^T^ containing acetyl muramic acid. Gram-staining was performed as described by Kamlage *et.al.* [54]. Acid fastness was tested by the methods described by Rohde [55]. Decomposition of purines, tyrosine and organic acids was tested using the method described by Gordon *et al.* [56]. Antibiotic susceptibility tests were performed with yeast malt agar using the method described previously [57]. Antibiotics were purchased from Bio-Rad, bioMérieux, Difco, BD and BBL.

Morphological characteristics of the strains were determined on cultures grown for 5–14 days on ISP2 agar (ISP2 containing additional 20 g l^−1^ agar) at 30 °C using light microscope (Imager M2, Carl Zeiss) and a field emission scanning electron microscope. Scanning electron microscopy was performed as described by Groth *et al.* [58]. Culture characteristics were determined on various ISP media for up to 18 days according to Shirling and Gottlieb [59] and similar to the approach described by Wink *et al.* [60]. Anaerobic and microaerophilic growth was tested by cultivating the strains at 28 °C in chambers with anaerobic or microaerophilic atmosphere generated by GENbox anaerob or GENbox microaer (bioMérieux cat. nos. 96124 and 96125). Colony colour was determined using Baumann’s Farbatlas 1 (Paul Baumann/Aue). Carbohydrate utilization was determined using ISP9 (carbon utilization medium) supplemented with 1 % sole carbon source. Melanoid pigment production was examined on peptone–yeast extract iron agar (ISP6), tyrosine agar (ISP7) and a synthetic medium from Suter [61] with and without tyrosine (1 g l^−1^). Sodium chloride tolerance was tested on ISP2 by changing sodium chloride concentrations from 1–15 %. The pH tolerance (pH range 4–10) was tested in ISP2 broth using a buffer system described by Xu *et al.* [62].

Whole-cell hydrolysates of RB20^T^ and RB56^T^ contained *meso*-diaminopimelic acid and the carbohydrates arabinose, galactose and traces of glucose. Free mycolic acids were present. The muramic acid of the peptidoglycan of both strains was glycosylated. Both strains were acid fast.

The predominant menaquinone MK-8 (H_4_, *ω*-cyclo) and small amounts of menaquinone MK-9(H_2_) were detected in both strains (Table 1).

**Table 1.** Physiological properties that separate the isolates from the type strains of phylogenetically close *Nocardia* species Strains: 1, RB20^T^; 2, *Nocardia miyunensis* JCM 12860^T^; 3, *Nocardia nova* DSM 44481^T^; 4, RB56^T^, 5, *Nocardia takedensis* DSM 44801^T^; 6, *Nocardia pseudobrasiliensis* DSM 44290^T^; 7, *Nocardia rayongensis* JCM 19832^T^. Data were taken from this study and previous studies [32–36]. Utilization tests were analyzed as followed: ++, grows better than positive control (basal medium with glucose); +, grows like positive control (basal medium with glucose); (+), better than negative control but not like positive control; −, not better than negative control (basal medium with water). All strains were positive for utilization of d-glucose and negative for utilization of raffinose and cellulose. Decomposition of purines, tyrosine and organic acids: −, no decomposition; (+), weak decomposition; +, decomposition; ++, very good decomposition.

Characteristics	1	2	3	4	5	6	7
DNA G+C content (mol%)	67.2	67.0	67.3	69.4	68.6	67.1	71.0
**Chemotaxonomic**							
Major menaquinone*	MK-8 (H_4_, ω-cyclo)	MK-8 (H_6_, ω-cyclo)^†^	MK-8 (H_4_, ω-cyclo)^‡^	MK-8* (H_4_, ω-cyclo)	MK-8 (H_4_, ω-cyclo)^§^	MK-8 (H_4_, ω-cyclo)^$^	MK-8 (H_4_, ω-cyclo)^¶^
Major fatty acids	C_16 : 0_, 10-methyl C_18 : 0_	C_16 : 0_, 10-methyl C_18 : 0_	C_16 : 0_, C_18 : 1_ *ω*9*c*	C_16 : 0_, C_18 : 1_ *ω*9*c*	C_16 : 0_, C_18 : 1_ *ω*9*c*	C_16 : 0_, C_18 : 1_ *ω*9*c*	C_16 : 0_, C_18 : 1_ *ω*9*c*
pH tolerance range for growth	5–7	4–8	4–8	5–7	6–8	5–9	4–7
Optimum pH for growth	6–7	6–7	6–7	6–7	6–7	6–7	6–7
Temperature growth range (°C)	15–37	15–37	15–37	15–37	15–37	15–45	15–37
Optimum temperature for growth (°C)	28	28	28	28	28	28	28
Anaerobic growth	−	−	−	−	−	−	−
Microaerophilic growth	+	+	+	(+)	+	+	+
Growth at NaCl concentration (% w/v)	0–3	0–3	0–7	0–1	0–3	0–9	0–3
**Utilization of sole carbon sources**							
Sucrose	(+)	+	(+)	(+)	−	−	−
d-Arabinose	+	+	+	+	−	−	−
d-Xylose	+	+	(+)	+	−	−	+
Inositol	+	−	−	−	−	+	−
d-Mannitol	+	++	−	−	−	+	(+)
d-Fructose	+	++	−	(+)	−	+	+
l-Rhamnose	+	−	−	+	−	−	−
**Decomposition of purines, tyrosine and organic acids**							
Citrate	−	(+)	−	−	−	+	(+)
Lactate	−	−	−	−	(+)	−	−
Acetate	−	(+)	+	(+)	−	(+)	(+)
Propionate	+	+	+	(+)	(+)	+	(+)
Malate	++	+	+	+	−	+	+
Pyruvate	(+)	+	−	(+)	+	+	(+)
Tyrosine	−	−	(+)	−	−	(+)	−
Adenine	−	−	+	−	−	+	−
Hypoxanthine	(+)	+	+	(+)	+	+	+
Xanthine	−	−	−	−	−	−	−

*Reference strains *N. asteroides* (DSM 43757, IMET 7547) [48, 49] and *N. carnae* (IMET 7504).

†Data from [32].

‡Data from [33].

§Data from [35].

¶Data from [36].

$Data from [34]

Strains RB20^T^ and RB56^T^ both exhibited similar polar lipid profiles with the major compounds diphosphatidylglycerol, phosphatidylethanolamine, phosphatidylinositol, phosphatidylinositol mannoside, two phospholipids (PL1, PL2) and two glycolipids (GL1, GL2). Strain RB20^T^ contained additional two unpolar lipids (L1, L2) and more polar lipid L3, which were not present in RB56^T^. In contrast, RB56^T^ revealed a third phospholipid (PL3) as well as two polar lipids (L4, L5), which is different from the lipids of RB20^T^ (Fig. S5).

The overall cellular fatty acid profiles of RB20^T^ and RB56^T^ were consistent with those of the genus *Nocardia* (Tables 1 and S7). The fatty acid profile of strain RB20^T^ was composed of the major fatty acids C_16 : 0_ (39.6 %), C_18 : 0_ 10-methyl (19.0 %), C_18 : 1_ *ω*9*c* (13.4 %) and C_18 : 0_ (9.9 %). The closest-related species, *N. miyunensis* JCM12860^T^ and *N. nova* DSM44481^T^, had similar fatty acid profiles and contained predominant amounts of C_16 : 0_ (39.5 and 38.3 %), C_18 : 0_ 10-methyl (18.8 and 14.7 %) and C_18 : 1_ *ω*9*c* (13.9 and 16.6 %).

In comparison, the fatty acid profile of strain RB56^T^ exhibited the major fatty acids C_16 : 0_ (42.8 %), C_18 : 1_ *ω*9*c* (16.1 %) and C_18 : 0_ 10-methyl (12.6 %) and minor amounts of C_14 : 0_ (6.9 %). The closest relative *N. takedensis* DSM44801^T^ was characterized by a relatively high amount of C_18 : 1_ω9*c* (27.4 %) and the presence of C_18 : 0_ (4.3 %) and C_20 : 1_ω9*c* (4.4 %) (Tables 1 and S7).

The following morphological and phenotypic characteristics were documented for strains RB20^T^ and RB56^T^, respectively (Table 1).

**Fig. 2.**
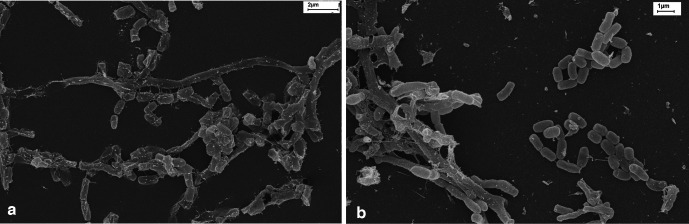
Scanning electron micrograph images of strain RB20^T^ (left) and strain RB56^T^ (right) cultivated at 28 °C on ISP3 agar for 14 days. Bars, 2 µm for RB20^T^ and 1 µm for RB56^T^.

Strain RB20^T^ grew at a pH range from pH 5 to 7 (optimum, pH 7) and at 0–3 % (w/v) NaCl (optimum, 0–1 %). Strain RB20^T^ tolerated a temperature range from 15 to 37 °C, but with only weak growth at 15 and 37 °C, and an optimal growth temperature of 28 °C. RB20^T^ showed fragmenting hyphae into short rod or coccoid forms (Fig. 2a).

Strain RB20^T^ grew well on ISP1–ISP7 and Suter medium. White aerial mycelium was well developed on ISP1–5 and ISP7. The substrate mycelium was beige-white on ISP1, ISP4 and ISP5, beige-orange on ISP2 and ISP3, and greyish ISP6 and Suter medium (Fig. S2). Overall, strain RB20^T^ exhibited different phenotypic characteristics compared to the reference strains *N. miyunensis* JCM12860^T^, *N. nova* DSM44481^T^ and *N. pseudobrasiliensis* DSM44290^T^ (Fig. S2, Table S6) with the following major differences: While RB20^T^ showed good growth on ISP2, ISP6 and Suter medium (+Tyr) and white-beige aerial and substrate mycelium, the closest relative *N. miyuensis* JCM 12860^T^ showed only very weak growth and ochre to orange aerial and substrate mycelium on ISP2 and ISP6, and good growth but ochre aerial and substrate mycelium on Suter medium (+Tyr). Similarly, RB20^T^ exhibited white-beige aerial and substrate mycelium on ISP7, while *N. nova* DSM 44481^T^ produced orange aerial and substrate mycelium.

Strain RB56^T^ grew at pH range 5–7 (optimum, pH 7.0) and at 0–1 % (w/v) NaCl (optimum, 0 %). Strain RB56^T^ tolerated a temperature range of 15–37 °C, with only weak growth at 15, 37 and 45 °C. The optimal growth temperature was 28 °C. On ISP2 medium, strain RB56^T^ formed short, round and ellipsoidal cells (Fig. 2b).

Strain RB56^T^ showed good growth on ISP2, ISP5 and ISP7, moderate growth on ISP1, ISP3, ISP4, and weak growth on ISP6 and Suter medium (Figs S3 and S4, Table S6). The substrate mycelium was orange on ISP1, ISP2, ISP6, ISP7 and Suter medium, white on ISP3, yellowish-white on ISP4 and orange-yellow on ISP5. White aerial mycelium developed on ISP3–5, white yellowish aerial mycelium on ISP7 and very poor orange aerial mycelium on ISP1 and ISP2. A soluble reddish pigment was observed on ISP7.

Overall, strain RB56^T^ exhibited different phenotypic characteristics to the reference strains *N. takedensis* DSM44801^T^, *N. pseudobrasiliensis* DSM44290^T^ and *N. rayongensis* JCM19832^T^ with the following major differences: While RB56^T^ showed good growth on ISP3 and white aerial and substrate mycelium, *N. takedensis* DSM 44801^T^ showed only weak growth and orange yellow substrate mycelium. Similarly, *N. pseudobrasiliensis* DSM 44290^T^ and *N. rayongensis* JCM 19832^T^ showed good growth on ISP4 with white aerial and substrate mycelium, whilst RB56^T^ and *N. takedensis* DSM 44801^T^ grew only moderately to weakly with yellowish to orange substrate mycelium. Finally, growth of RB56^T^ was only weak on Suter medium (with/without tyrosine), whilst all reference strains grew well showing orange to brown soluble pigmentation.

Both strains, RB20^T^ and RB56^T^, were resistant to oxytetracycline, azlocillin, lincomycin, trimethoprim, carbenicillin, piperacillin, cefoxitin, mezlocillin, penicillin G, cephalothin and chlortetracycline.

Strain RB20^T^ was furthermore resistant against tetracycline and novobiocin, whereas RB56^T^ exhibited resistance against polymyxin B and erythromycin (Table S8).

The morphological, physiological, genetic and chemotaxonomic data support the delineation of RB20^T^ and RB56^T^ as two novel species of the genus *Nocardia *.

## Description of *Nocardia macrotermitis* sp. nov.

*Nocardia macrotermitis* (ma.cro.ter´mi.tis. N.L. gen. n. *macrotermitis*, of the termite *Macrotermes*, from where the organism was first isolated).

Cells are Gram-stain-positive, aerobic and acid-fast. Colonies form branched vegetative mycelium that fragment into short rod and coccoid forms. Good growth occurs on all media tested within 12 days. Aerial mycelium is formed on ISP2, ISP3, ISP5, ISP7 and Suter and poorly on ISP1, ISP4 and ISP6.

The pH range for growth is pH 5–7. Growth temperature range is between 15–45 °C with optimum growth at 28 °C. The maximum concentration of NaCl for growth is 3 % (w/v). The strain grows on the following sole carbon sources: glucose, arabinose, xylose, inositol, mannitol, fructose and rhamnose; but does not grow with raffinose and cellulose.

The strain is able to utilize the organic acids propionate, malate and decomposes pyruvate and hypoxanthine weakly. Resistant to oxytetracycline, azlocillin, lincomycin, tetracycline, chlortetracycline, trimethoprim, carbenicillin, piperacillin, cefoxitin, mezlocillin, penicillin G, cephalothin and novobiocin.

The diagnostic diamino acid of the cell wall is *meso*-diaminopimelic acid (*meso*-A_2_pm). Mycolic acids and *N*-glycosylmuramic acid in the glycan part of the peptidoglycan are present.

The sugars in whole-cell hydrolysates are arabinose, galactose and traces of glucose. Major polar lipids are diphosphatidylglycerol, phosphatidylethanolamine, phosphatidylinositol, phosphatidylinositol mannoside, two phospholipids (PL1, PL2) and two glycolipids (GL1, GL2). Furthermore, two unpolar lipids (L1, L2) and a polar lipid (L3) are present. The predominant menaquinone is MK-8(H_4_, *ω*-cyclo). The major fatty acids are hexadecanoic acid (C_16 : 0_), 10-methyl octadecanoic acid (10-methyl C_18 : 0_), *cis-*9-octadecenoic acid (C_18 : 1_ ω9*c*) and octadecanoic acid (C_18 : 0_). The DNA G+C content of strain RB20^T^ is 67.2 mol%.

The type strain, RB20^T^ (=VKM Ac-2841^T^=NRRL-B65541^T^), was isolated from the gut of the termite *Macrotermes natalensis* (major worker). The strain has been deposited in the All-Russian Collection of Microorganisms (=VKM Ac-2841^T^) and the Agricultural Research Service Culture Collection (=NRRL B65541^T^). The GenBank/EMBL accession number for the partial 16S rRNA gene sequence is KY558706.2. This Whole Genome Shotgun project has been deposited at DDBJ/ENA/GenBank under the accession WEGK00000000 (Bio project PRJNA545686, Biosample SAMN11902338). The version described in this paper is version WEGK01000000.

## Description of *Nocardia aurantia* sp. nov.

*Nocardia aurantia* (au.ran'ti.a. N.L. fem. adj. *aurantia*, orange-coloured, referring to the gold-coloured substrate mycelium).

Cells are Gram-stain-positive, aerobic and acid-fast. Colonies form branched vegetative mycelium that fragment into short rod and coccoid forms. Good growth occurs on ISP2, ISP5 and ISP7, moderate growth on ISP1, ISP3 and ISP4, and poor growth on ISP6 and Suter medium. Aerial mycelium is formed on ISP3, ISP4 and ISP7 media, very poorly on ISP1, ISP2 and ISP5, but not at all on ISP6 or Suter medium. Short, round, ellipsoidal spores are formed. A reddish pigment is produced on ISP7.

The pH range for growth is pH 5–7. The growth temperature range is 15–37 °C with optimal growth at 28 °C. The maximum concentration of NaCl for growth is 1 % (w/v). The strain grows on the following sole carbon sources: glucose, arabinose, xylose and rhamnose; but not with raffinose, cellulose, inositol or mannitol.

The strain is able to weakly utilize the organic acids acetate, propionate, pyruvate and hypoxanthine, and decomposes malate. Resistant to oxytetracycline, azlocillin, lincomycin, trimethoprim, carbenicillin, piperacillin, cefoxitin, mezlocillin penicillin G, cephalothin, chlortetracycline, polymyxin B and erythromycin.

The diagnostic diamino acid of the cell wall is *meso*-diaminopimelic acid (*meso*-A_2_pm). Mycolic acids and *N*-glycosylmuramic acid in the glycan part of the peptidoglycan are present.

Whole-cell hydrolysates contain arabinose, galactose and traces of glucose. Major polar lipids are diphosphatidylglycerol, phosphatidylethanolamine, phosphatidylinositol, phosphatidylinositol mannoside, three unidentified phospholipids (PL1, PL2 and PL3), two unknown lipids and two glycolipids (GL1, GL2). The predominant menaquinone is MK-8(H_4_, ω-cyclo). The major fatty acids are hexadecaonic acid (C_16 : 0_), *cis*-9-octadecenoic acid (C_18 : 1_ *cis*-9) and 10-methyl octadecanoic acid (10-methyl C_18 : 0_). The DNA G+C content of strain RB56^T^ is 69.4 mol%.

The type strain, RB56^T^ (=VKM Ac-2842^T^=NRRL-B65542^T^), was isolated from the gut of the termite *Macrotermes natalensis* (major worker). The strain has been deposited in the All-Russian Collection of Microorganisms (=VKM Ac-2842^T^) and the Agricultural Research Service Culture Collection (=NRRL B65542^T^). The GenBank/EMBL accession number for the partial 16S rRNA gene sequence is KY558730.2. This Whole Genome Shotgun project has been deposited at DDBJ/ENA/GenBank under the accession WEGI00000000 (Bio project PRJNA545686, Biosample SAMN11902338). The version described in this paper is version WEGI01000000.

## Supplementary Data

Supplementary material 1
