# Supplementary material for: Nocardia macrotermitis sp. nov. and Nocardia aurantia sp. nov., isolated from the gut of the fungus-growing termite Macrotermes natalensis
Source: Int J Syst Evol Microbiol. 2020 Aug 20;70(10):5226–34. doi: 10.1099/ijsem.0.004398 (PMC7660896; doi:10.1099/ijsem.0.004398)
Supplement: Supplementary material 1 [file ijsem-70-5226-s001.pdf]

Nocardia macrotermitis* sp. nov. and *Nocardia aurantia* sp. nov., isolated from the gut of the fungus-growing termite *Macrotermes natalensis

René Benndorf[1*], Jan W. Schwitalla[1*], Karin Martin[1*], Wilhelm de Beer[2], John Vollmers[3], Anne-Kristin Kaster[3], Michael Poulsen,[4] and Christine Beemelmanns[1*]

[1]Leibniz Institute for Natural Product Research and Infection Biology e. V., Hans-Knöll-Institute, Beutenbergstraße 11a, 07745 Jena, Germany

[2]Department of Microbiology and Plant Pathology, Forestry and Agriculture Biotechnology Institute, University of Pretoria, 00028 Hatfield, South Africa

[3]Institute for Biological Interfaces (IBG 5), Karlsruhe Institute of Technology, Hermann-von- Helmholtz-Platz 1, 76344 Eggenstein-Leopoldshafen, Germany

[4]University of Copenhagen, Department of Biology, Section for Ecology and Evolution, Universitetsparken 15, 2100 Copenhagen East, Denmark

Correspondence

Christine Beemelmanns, E-mail: christine.beemelmanns@leibniz-hki.de

Karin Martin, E-mail: karin.martin@leibniz-hki.de

#Authors contributed equally to the manuscript

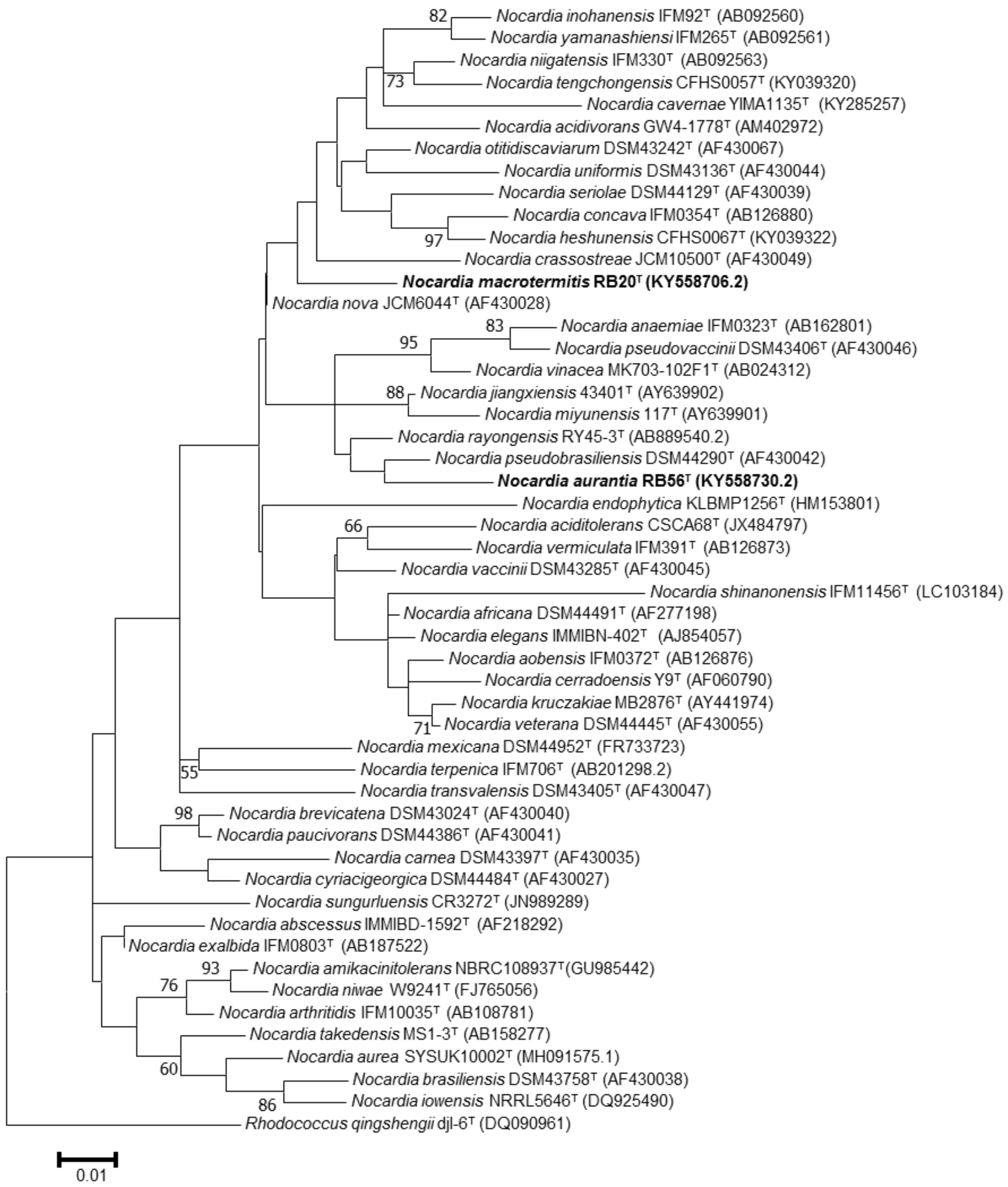

Figure S1. Maximum-likelihood phylogenetic tree showing the phylogenetic relationship of strain RB20^{T}, RB56^{T} and other closely related species based on 16S rRNA gene sequences. *Rhodococcus qingshengii* djl-6T was used to root the tree. Only bootstrap values above 50% (1000 pseudoreplications) are shown. Bar length corresponds to 0.01 substitutions per nucleotide position.

Table S1. Calculated sequence similarities based on nearly full length 16S rRNA gene of strain RB20^T and *Nocardia* reference strains.

**Strain (Accession number)**	**Similarity [%]**
Nocardia miyunensis 117^T (AY639901)	98.93
Nocardia nova JCM6044^T (AF430028)	98.52
Nocardia niigatensis IFM330^T (AB092563)	98.36
Nocardia pseudobrasiliensis DSM44290^T (AF430042)	98.32
Nocardia jiangxiensis 43401^T (AY639902)	98.15
Nocardia vaccinii DSM43285^T (AF430045)	98.11
Nocardia rayongensis RY45-3^T (AB889540.2)	98.10
Nocardia tengchongensis CFHS0057^T (KY039320)	98.06
Nocardia yamanashiensis IFM265^T (AB092561)	98.06

[a] Sequence similarities were calculated using the method recommended by Meier-Kolthoff [1].

Table S2. Calculated sequence similarities based on nearly full length 16S rRNA gene of strain RB56^T and *Nocardia* reference strains.

**Strain (Accession number)**	**Similarity [%]**
Nocardia takedensis MS1-3^T (AB158277)	98.34
Nocardia pseudobrasiliensis DSM44290^T (AF430042)	98.31
Nocardia rayongensis RY45-3^T (AB889540.2)	98.24
Nocardia nova JCM6044^T (AF430028)	98.18
Nocardia iowensis NRRL5646^T (DQ925490)	98.17
Nocardia elegans IMMIBN-402^T (AJ854057)	98.17
Nocardia kruczakiae MB2876^T (AY441974)	98.04

[b]Sequence similarities were calculated using the method recommended by Meier-Kolthoff [1]

Table S3. Digital DDH values of RB20^{T} and *Nocardia* genomes available at NCBI server and RB56^{T}.

Strain (Accession number)	NCBI number	Similarity[e] [%]
Nocardia jiangxiensis NBRC101359^{T}	BAGB00000000.1	34.3
Nocardia miyunensis NBRC108239^{T}	BDBQ00000000.1	33.9
Nocardia vaccinii NBRC15922^{T}	BDCC00000000.1	32.4
Nocardia terpenica NBRC 100888^{T}	GCA_000320925.1	24.7
Nocardia mexicana NBRC108244^{T}	BDBV00000000.1	23.0
Nocardia transvalensis NBRC15921^{T}	BAGL00000000.1	23.0
Nocardia pseudobrasiliensis NBRC108224^{T}	BDBS00000000.1	22.6
Nocardia cerradoensis NBRC101014^{T}	BAFW00000000.1	22.4
Nocardia veterana NBRC100344^{T}	BAGM00000000.1	22.4
Nocardia mikamii NBRC108933^{T}	BDCM00000000.1	22.3
Nocardia africana NBRC100379^{T}	BDAV00000000.1	22.2
Nocardia aobensis NBRC100429^{T}	BAFQ00000000.1	22.2
Nocardia kruczakiae NBRC101016^{T}	BDBL00000000.1	22.2
Nocardia violaceofusca NBRC14427^{T}	BDCN00000000.1	22.1
Nocardia elegans NBRC108235^{T}	BDBF00000000.1	22.0
Nocardia nova NBRC15556^{T}	BDBN00000000	22.0
RB56^{T}		21.9
Nocardia otitidiscaviarum NBRC14405^{T}	BAGD00000000.1	21.9
Nocardia xishanensis NBRC101358^{T}	BDCF00000000.1	21.7
Nocardia concava NBRC100430^{T}	BAFX00000000.1	21.6
Nocardia pneumoniae NBRC100136^{T}	BAGF00000000.1	21.6
Nocardia puris NBRC108233^{T}	BDBW00000000.1	21.6
Nocardia tenerifensis NBRC101015^{T}	BAGH00000000.1	21.6
Nocardia abscessus NBRC 100374^{T}	BAFP00000000.1	21.5
Nocardia amamiensis NBRC102102^{T}	BDBA00000000.1	21.5
Nocardia arthritidis NBRC100137^{T}	BDBB00000000.1	21.5
Nocardia crassostreae NBRC100342^{T}	BDCH00000000.1	21.5
Nocardia farcinica NCTC11134^{T}	LN868938.1	21.5
Nocardia lijiangensis NBRC108240^{T}	BDBP00000000.1	21.5
Nocardia niigatensis NBRC100131^{T}	BAGC00000000.1	21.5
Nocardia amikacinitolerans NBRC108937^{T}	BDAU00000000.1	21.4
Nocardia asiatica NBRC100129^{T}	BAFS00000000.1	21.4
Nocardia exalbida NBRC100660^{T}	BAFZ00000000.1	21.4
Nocardia gamkensis NBRC108242^{T}	BDBM00000000.1	21.4
Nocardia jejuensis NBRC103114^{T}	BDBU00000000.1	21.4
Nocardia vermiculata NBRC100427^{T}	BDCA00000000.1	21.4
Nocardia anaemiae NBRC100462^{T}	BDAZ00000000.1	21.3
Nocardia araoensis NBRC100135^{T}	BAFR00000000.1	21.3
Nocardia brasiliensis NBRC 14402^{T}	ASM30847v2	21.4
Nocardia cyriacigeorgica NBRC100375^{T}	ASM30855v1	21.4
Nocardia higoensis NBRC100133^{T}	BAGA00000000.1	21.3
Nocardia niwae NBRC108934^{T}	BDCK00000000.1	21.3
Nocardia pseudovaccinii NBRC100343^{T}	BDBY00000000.1	21.3
Nocardia seriolae NBRC15557^{T}	ASM799071v1	21.3
Nocardia uniformis NBRC13702^{T}	BDCE00000000.1	21.3
Nocardia vinacea NBRC16497^{T}	BAGN00000000.1	21.3
Nocardia yamanashiensis NBRC100130^{T}	BDCD00000000.1	21.3

Table S4. Digital DDH values of RB56^{T} and *Nocardia* genomes available at NCBI server.

Strain (Accession number)	NCBI number	Similarity[c] [%]
Nocardia terpenica NBRC 100888^{T}	GCA_000320925.1	24.7
Nocardia mexicana NBRC108244^{T}	BDBV00000000.1	22.8
Nocardia transvalensis NBRC15921^{T}	BAGL00000000.1	22.6
Nocardia pseudobrasiliensis NBRC108224^{T}	BDBS00000000.1	22.3
Nocardia cerradoensis NBRC101014^{T}	BAFW00000000.1	22.0
Nocardia miyunensis NBRC108239^{T}	BDBQ00000000.1	22.0
Nocardia veterana NBRC100344^{T}	BAGM00000000.1	22.0
Nocardia aobensis NBRC100429^{T}	BAFQ00000000.1	21.9
Nocardia vaccinii NBRC15922^{T}	BDCC00000000.1	21.9
Nocardia africana NBRC100379^{T}	BDAV00000000.1	21.8
Nocardia elegans NBRC108235^{T}	BDBF00000000.1	21.8
Nocardia jiangxiensis NBRC101359^{T}	BAGB00000000.1	21.8
Nocardia kruczakiae NBRC101016^{T}	BDBL00000000.1	21.8
Nocardia nova NBRC15556^{T}	BDBN00000000	21.8
Nocardia violaceofusca NBRC14427^{T}	BDCN00000000.1	21.8
Nocardia farcinica NCTC11134^{T}	LN868938.1	21.7
Nocardia mikamii NBRC108933^{T}	BDCM00000000.1	21.7
Nocardia otitidiscaviarum NBRC14405^{T}	BAGD00000000.1	21.6
Nocardia pneumoniae NBRC100136^{T}	BAGF00000000.1	21.6
Nocardia asiatica NBRC100129^{T}	BAFS00000000.1	21.5
Nocardia amikacinitolerans NBRC108937^{T}	BDAU00000000.1	21.4
Nocardia amamiensis NBRC102102^{T}	BDBA00000000.1	21.4
Nocardia concava NBRC100430^{T}	BAFX00000000.1	21.4
Nocardia exalbida NBRC100660^{T}	BAFZ00000000.1	21.4
Nocardia lijiangensis NBRC108240^{T}	BDBP00000000.1	21.4
Nocardia niigatensis NBRC100131^{T}	BAGC00000000.1	21.4
Nocardia puris NBRC108233^{T}	BDBW00000000.1	21.4
Nocardia tenerifensis NBRC101015^{T}	BAGH00000000.1	21.4
Nocardia xishanensis NBRC101358^{T}	BDCF00000000.1	21.4
Nocardia araoensis NBRC100135^{T}	BAFR00000000.1	21.3
Nocardia arthritidis NBRC100137^{T}	BDBB00000000.1	21.3
Nocardia cyriacigeorgica NBRC100375^{T}	ASM30855v1	21.3
Nocardia higoensis NBRC100133^{T}	BAGA00000000.1	21.3
Nocardia seriolae NBRC15557^{T}	ASM799071v1	21.2
Nocardia abscessus NBRC100374^{T}	BAFP00000000.1	21.2
Nocardia beijingensis NBRC16342^{T}	BDBC00000000.1	21.2
Nocardia jejuensis NBRC103114^{T}	BDBU00000000.1	21.2
Nocardia niwae NBRC108934^{T}	BDCK00000000.1	21.2
Nocardia vulneris NBRC108936^{T}	BDCI00000000.1	21.2
Nocardia brasiliensis NBRC 14402^{T}	ASM30847v2	21.3
Nocardia gamkensis NBRC108242^{T}	BDBM00000000.1	21.1
Nocardia yamanashiensis NBRC100130^{T}	BDCD00000000.1	21.1
Nocardia altamirensis NBRC108246^{T}	BDAY00000000.1	21.0
Nocardia crassostreae NBRC100342^{T}	BDCH00000000.1	21.0
Nocardia shimofusensis NBRC100134^{T}	BDBT00000000.1	21.0
Nocardia brevicatena NBRC12119^{T}	BAFU00000000.1	20.9
Nocardia flavorosea NBRC108225^{T}	BDCG00000000.1	20.9
Nocardia takedensis NBRC 100417^{T}	BAGG00000000.1	20.7

[c] Digital DDH values were calculated using the GGDC web server available at http://ggdc.dsmz.de/ [2]

Table S5. Summary of *Nocardia* genomes sequenced in this work, including strain ID, genus, total genome size (in mega base pairs), GC content in %, number of assembled contigs (contiguous sequences).

**Strain ID**	**RB20**	**RB56**
Genus	*Nocardia*	*Nocardia*
Total size [Mb]	~8.6	~8.6
GC content [%]	67.2	69.4
Number of contigs	60	67
N50 [bp]	425.626	451.059
L50	7	8
Total CDS	7454	7605
Estimated completeness [%]	98.99	99.70
Estimated contamination[b]	1.80	1.74

b Contamination=Fraction [%] of identified universal marker genes that occur in multiple copy number (does not necessarily indicate actual contamination)

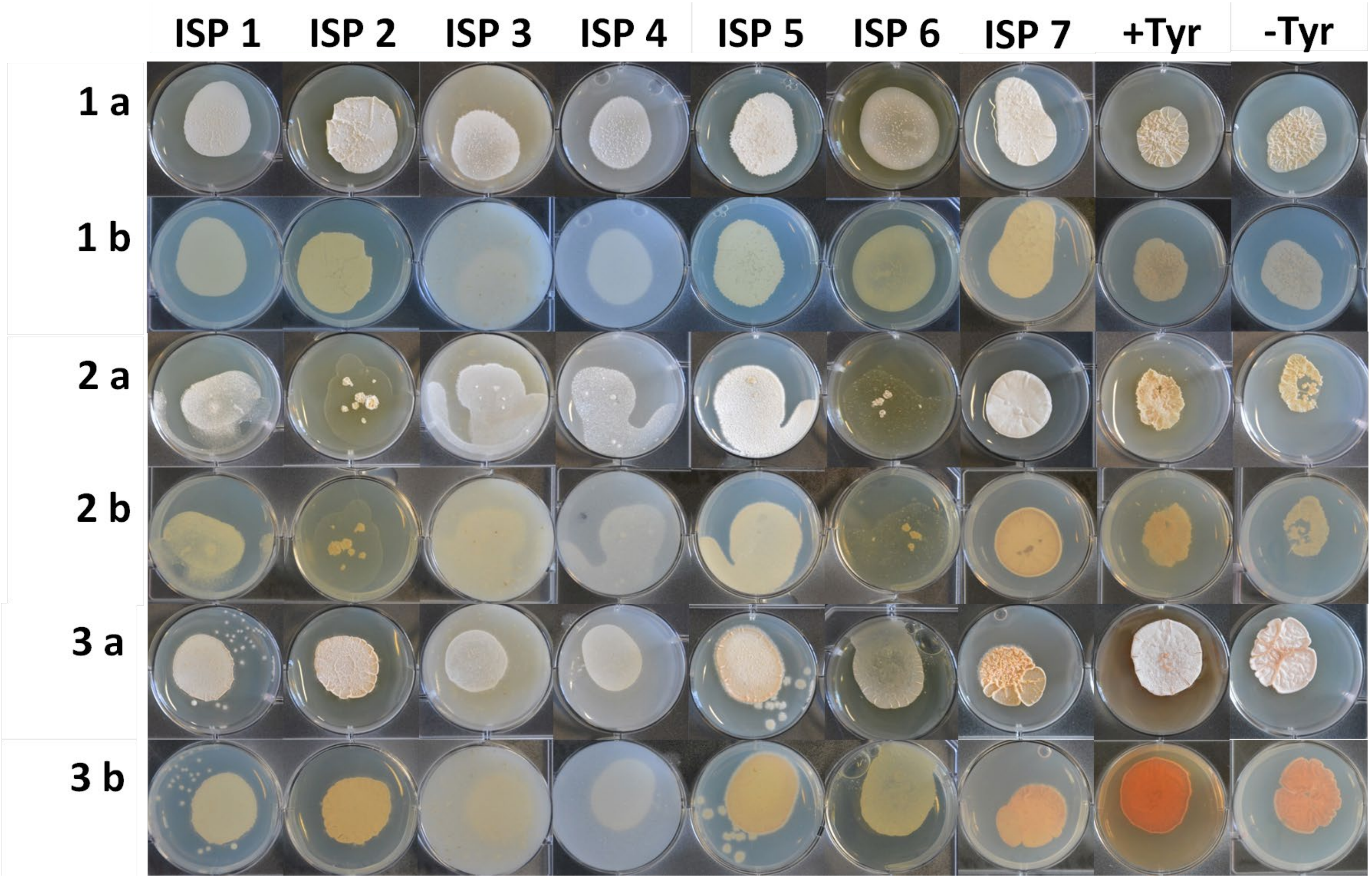

Figure S2. Morphology of strains grown on different ISP-media and Suter-Medium (with: +Tyr [1 g/L] and without tyrosine: -Tyr) for 12 days at 28 °C.

Strains: RB20^T (1a: above, 1b: reverse); *Nocardia miyuensis* JCM 12860^T (2a: above, 2b: reverse); *Nocardia nova* DSM 44481^T (3a: above; 3b: reverse).

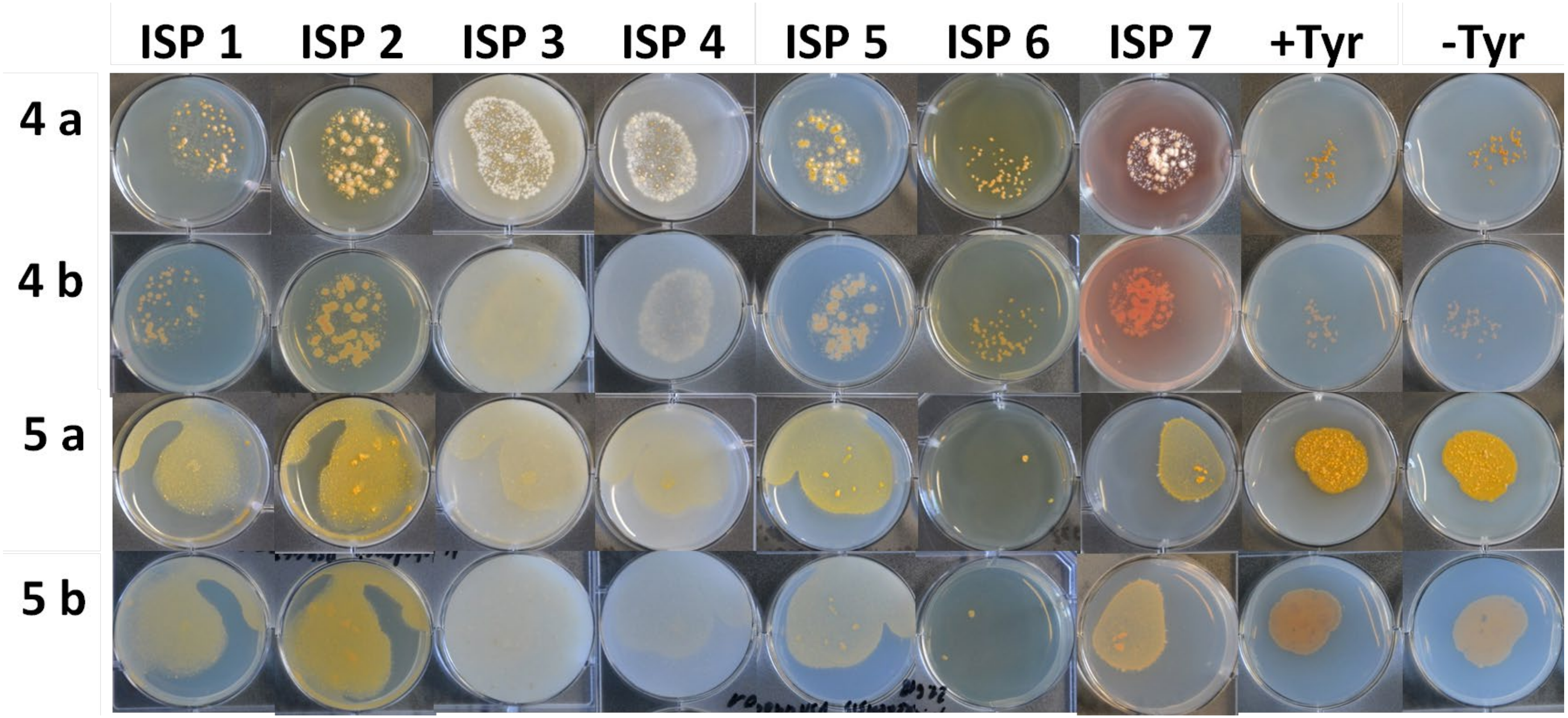

Figure S3. Morphology of strains grown on different ISP-media and Suter-Medium (with: +Tyr [1 g/L] and without tyrosine: -Tyr) for 12 days at 28°C.

Strains: RB56^{T} (4a: above, 4b: reverse); *Nocardia takedensis* DSM 44801^{T} (5a: above, 5b: reverse)

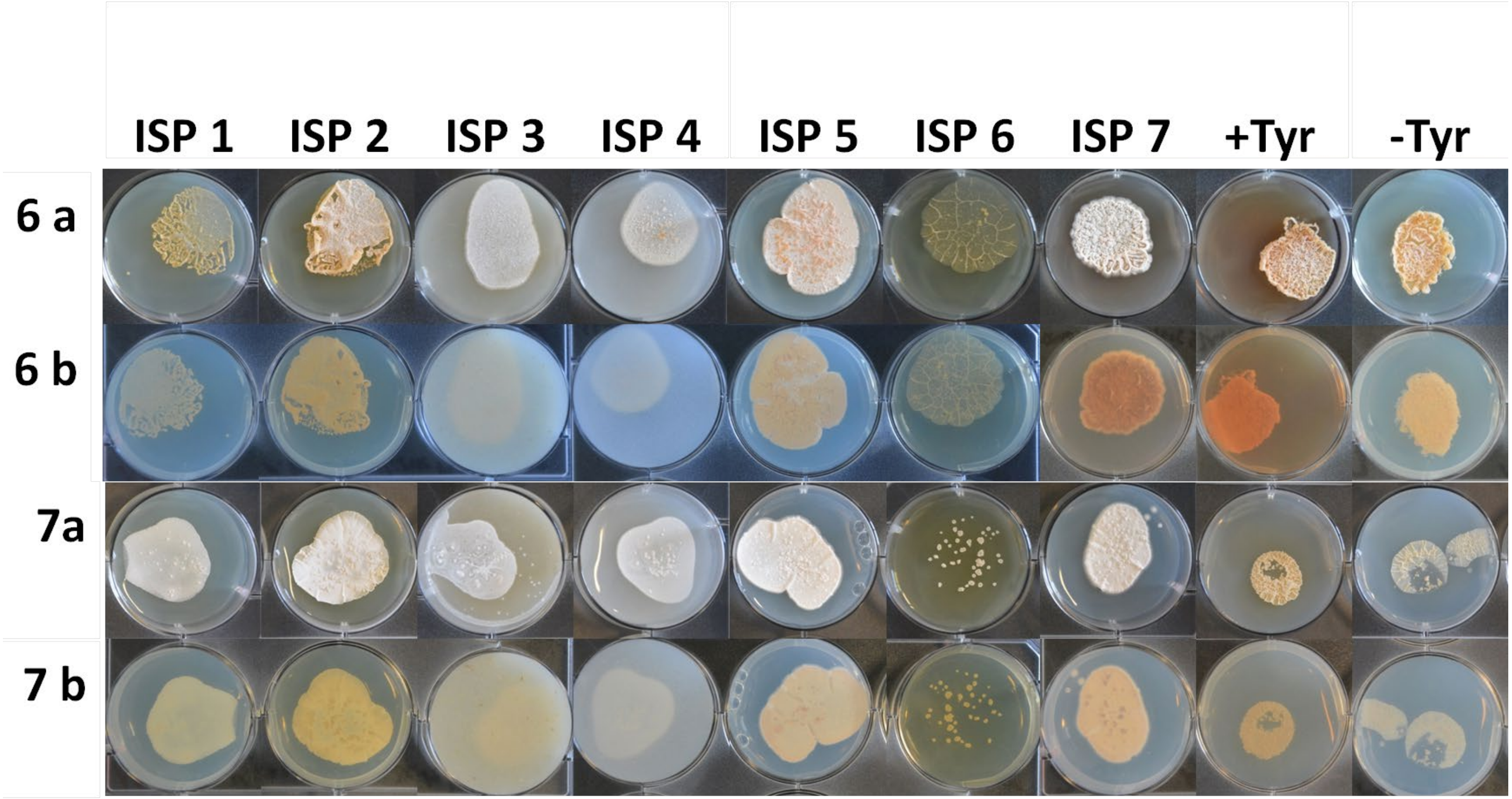

Figure S4. Morphology of strains grown on different ISP-media and Suter-Medium (with: +Tyr [1 g/L] and without tyrosine: -Tyr) for 12 days at 28 °C.

Strains: *Nocardia pseudobrasiliensis* DSM 44290^{T} (6a: above, 6b: reverse); *Nocardia rayongensis* JCM 19832^{T} (5a: above, 5b: reverse)

Table S6. Cultural characteristics of strains RB20^T and RB56^T and type strains of closely related *Nocardia* species after 12-14 days of incubation at 28 °C. Strains: 1. RB20^T; 2. *N. miyunensis* JCM 12860^T, 3. *N. nova* DSM 44481^T, 4. RB56^T, 5. *N. takedensis* DSM 44801^T, 6. *N. pseudobrasiliensis* DSM 44290^T, 7. *N. rayongensis* JCM 19832^T. All data were acquired in this study. Morphological feature: G growth, AM aerial mycelium, SM substrate mycelium, SP soluble pigment, Colour coding (No.) corresponding to Baumanns Farbatlas 1 in parentheses.

**Medium**	**Morphol. feature**	**1**	**2**	**3**	**4**	**5**	**6**	**7**
ISP1	G	Good	Good	Good	Moderate	Good	Good	Good
	AM	Poor, white	White	White	Very poor, Orange (No. 104)	None	None	White
	SM	White	White	Whitish ocher (No.101-103)	Orange (No.104)	Orange-yellow (No.71)	Orange yellow (No.71)	Whitish ocher (No.93-94)
	SP	None	None	None	None	None	None	None
ISP2	G	Good	Weak	Good	Good	Good	Good	Good
	AM	White	White	White	Very poor, Orange (No. 104)	None	Whitish orange (No.98)	White
	SM	Beige	Orange	Orange (No.104)	Orange	Orange-yellow (No.71)	orange	Whitish ocher (No.93-94)
	SP	None	None	None	None	None	None	None
ISP3	G	Good	Moderate	Good	Moderate	Weak	Good	Good
	AM	White	White	White	White	White	White	White
	SM	White	White	White	White	Orange-yellow (No.71)	Beige	Beige
	SP	None	None	None	None	None	None	None
ISP4	G	Good	Moderate	Good	Moderate	Weak	Good	Good
	AM	Poor, white	None	Very poor, white	White	None	Poor on margin, white	White
	SM	White	White	White	White yellowish (No.94)	Orange-yellow (No.71)	White	White
	SP	None	None	None	None	None	None	None

ISP5	G	Good	Good	Good	Good	Good	Good	Good
	AM	White	Poor on margin, white	Very poor on margin, white	White	none	White	White
	SM	Beige	Beige	Light orange (No.104)	Orange-yellow (No.71)	Orange-yellow (No.71)	Orange	Whitish ocher (No.93-94)
	SP	None	None	None	None	None	None	None
ISP6	G	Good	Weak	Good	Weak	Poor	Moderate	Weak
	AM	Very poor, white	Ochre (No.102)	Very poor, white	None	None	None	White
	SM	Greyish	Orange (No. 104)	Light yellow (No.11)	Orange (No.104)	Orange-yellow (No.71)	Orange Yellow (No.71)	Whitish ocher (No.93-94)
	SP	None	None	None	None	None	None	None
ISP7	G	Good	Good	Good	Good	Moderat e	Good	Good
	AM	White	None	Poor on margin	White yellowish (No.94)	Poor on margin	White	White
	SM	Yellow-beige	Orange	Orange	Orange	Orange-yellow (No.71)	Orange	Orange
	SP	None	None	Orange (No.104)	Reddish pigment (No.216)	None	None	None
Suter medium without tyrosine	G	Good	Good	Good	Weak	Good	Good	Moderate
	AM	White	White	White	None	None	White	None
	SM	Beige-orange	Ocher (No.102)	Dark orange	Orange (No.105)	Orange-yellow (No.71)	Whitish orange (No.98)	whitish ocher (No.93-94)
	SP	None	None	None	None	None	None	None
Suter medium with tyrosine	G	Good	Good	Good	Weak	Good	Good	Moderate
	AM	None	Ocher (No.102)	White	None	None	White	None
	SM	White	Ocher (No.102)	White	Orange	Orange-yellow (No.71)	Whit-ish orange (No.98)	Ocher (No.102)
	SP	None	Greenish brown (No.114)	Brown (No.173)	None	Brown (No.117)	Brown (No.173)	Greenish brown (No.114)

Table S7. Cellular fatty acid compositions (in %) of strains RB20^T and RB56^T and type strains of closely related *Nocardia* species: 1. RB20^T; 2. *N. miyunensis* JCM12860^T; 3. *N. nova* DSM44481^T 4. RB56^T; 5. *N. takedensis* DSM44801^T, 6. *N. pseudobrasiliensis* DSM44290^T, 7. *N. rayongensis* JCM19832^T. Data below 1.0% were marked with tr; traces and not detected fatty acids are marked with -. All data were acquired in this study.

**Fatty acids**	**1**	**2**	**3**	**4**	**5**	**6**	**7**
Saturated fatty acids							
14:0	tr	tr	tr	6.9	1.6	tr	tr
15:0	tr	tr	tr	tr	tr	1.71	2.11
16:0	39.6	39.5	38.3	42.82	40.0	41.4	39.5
17:0	tr	-	1.9	-	tr	1.9	2.2
17:0 10-methyl	tr	-	-	-	-	tr	1.1
18:0	9.9	3.3	7.1	tr	4.3	tr	tr
18:0 10-methyl (TSBA)	19.0	18.8	14.7	12.6	8.3	13.7	15.2
Unsaturated fatty acids							
17:1 *ω5cis*	tr	1.0	-	-	-	-	tr
17:1 *ω8cis*	tr	-	-	-	tr	2.3	2.5
18:1 *ω9cis*	13.4	13.9	16.6	16.1	27.4	19.9	17.2
20:1 *ω9cis*	tr	4.0	tr	-	4.5	-	-
Branched fatty acids							
iso-16:0	1.5	tr	-	1.6	-	-	-
Summed feature3[a]	9.2	9.0	17.7	16.8[b]	8.8	16.9	18.7

[a]composition: 15:0 ISO 2OH/16:1 *ω6cis/*16:1 *ω7cis*; [b]composition for RB56:15:0 ISO 2OH/16:1 *ω6cis/*16:1 *ω5cis*

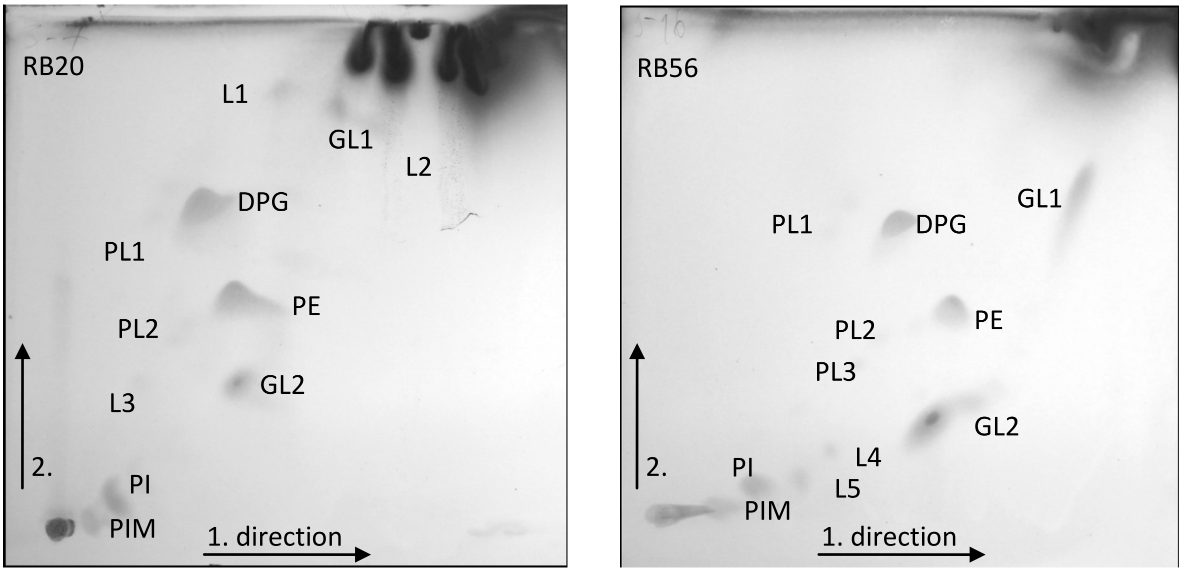

Fig S5. Polar lipid profile of strains RB20^T and RB56^T after two-dimensional TLC and detection with molybdatophosphoric acid. Arrows indicate first and second development of the TLC. The solvent system consisted of chloroform-methanol-water (65:25:4, by vol.) for the first development, and of chloroform-methanol-glacial acetic acid-water (80:12:15:4, by vol.) for second development. DPG, diphosphatidylglycerol; PE, phosphatidylethanolamine; PI, phosphatidylinositol; PIM, phosphatidylinositol mannoside; PL1-PL3, unknown phospholipids; GL1, GL2, unknown glycolipids; L1,-L5, unknown lipids.

Table S8. Antibiotic susceptibility test of strains RB20^T and RB56^T and type strains of closely related *Nocardia* species after 12-14 days of incubation at 28 °C.[1] The diameter of the inhibition zone is given in mm. Strains: 1. RB20^T; 2. *N. miyunensis* JCM 12860^T, 3. *N. nova* DSM 44481^T, 4. RB56^T, 5. *N. takedensis* DSM 44801^T, 6. *N. pseudobrasiliensis* DSM 44290^T, 7. *N. rayongensis* JCM 19832^T All strains are not susceptible to oxytetracyclin, azlocillin, lincomycin, trimethoprim, carbenicillin, piperacillin, cefoxitin, mezlocillin.

Antibiotic	Conc.	1	2	3	4	5	6	7
Penicillin G	10 units	0	14	0	0	11	0	12
Vancomycin	30 µg	8	30	20	24	26	18	24
Imipenem	10 µg	62	40	35	40	55	18	40
polymyxin B	30 units	12	12	10	0	10	18	10
Amoxicillin + clavulanic acid	20, 10 µg	12	20	0	0	0	0	15
Amikacin	30 µg	48	30	25(40)	64	35	42	30
Cephalothin	30 µg	0	20	0	0	0	0	17
Bacitracin	130 µg	16	12	16	20	32	22	25
Ampicillin	10 µg	22	18	0	18	18	0	25
Kanamycin	30 µg	25	30	24	20	28	22	26
Tetracyclin	30 µg	0	20	0	14	0	10	18
Chloramphenicol	30 µg	30	30	28	20	0	20	30
Streptomycin	10 µg	38	50	24	18	22	0	50
Chlortetracyclin	30 µg	0	20	0	0	10	0	16
Rifampin	5 µg	34	40	15	26	18	14	40
Gentamycin	10 µg	14	24	12	12	18	30	30
Erythromycin	15 µg	45	46	34	0	17	32	40
Doxycycline	30 µg	19	24	14	0	21	9	22
Ciprofloxacin	5 µg	26	30	15	24	0	55	30
Norfloxacin	10 µg	14	30	0	18	0	34	20
Novobiocin	5 µg	0	0	15	16	0	0	0

[1] 50. Groth I, Schütze B, Boettcher T, Pullen CB, Rodriguez C, Leistner E, et al. *Kitasatospora putterlickiae* sp. nov., isolated from rhizosphere soil, transfer of *Streptomyces kifunensis* to the genus *Kitasatospora* as *Kitasatospora kifunensis* comb. nov., and emended description of *Streptomyces aureofaciens* Duggar 1948. 2003;53(6):2033-2040. DOI: 10.1099/ijs.0.02674-0
